# Prenatal Exome Sequencing: Background, Current Practice and Future Perspectives—A Systematic Review

**DOI:** 10.3390/diagnostics11020224

**Published:** 2021-02-02

**Authors:** Daniele Guadagnolo, Gioia Mastromoro, Francesca Di Palma, Antonio Pizzuti, Enrica Marchionni

**Affiliations:** 1 Department of Experimental Medicine, Policlinico Umberto I Hospital, Sapienza University of Rome, 00161 Rome, Italy; daniele.guadagnolo@uniroma1.it (D.G.); gioia.mastromoro@uniroma1.it (G.M.); francesca.dipalma@uniroma1.it (F.D.P.); antonio.pizzuti@uniroma1.it (A.P.); 2 Clinical Genomics Unit, IRCCS Casa Sollievo della Sofferenza, 71013 San Giovanni Rotondo (FG), Italy

**Keywords:** prenatal exome sequencing, pES, NGS, prenatal diagnosis, systematic review, ES

## Abstract

The introduction of Next Generation Sequencing (NGS) technologies has exerted a significant impact on prenatal diagnosis. Prenatal Exome Sequencing (pES) is performed with increasing frequency in fetuses with structural anomalies and negative chromosomal analysis. The actual diagnostic value varies extensively, and the role of incidental/secondary or inconclusive findings and negative results has not been fully ascertained. We performed a systematic literature review to evaluate the diagnostic yield, as well as inconclusive and negative-result rates of pES. Papers were divided in two groups. The former includes fetuses presenting structural anomalies, regardless the involved organ; the latter focuses on specific class anomalies. Available findings on non-informative or negative results were gathered as well. In the first group, the weighted average diagnostic yield resulted 19%, and inconclusive finding rate 12%. In the second group, the percentages were extremely variable due to differences in sample sizes and inclusion criteria, which constitute major determinants of pES efficiency. Diagnostic pES availability and its application have a pivotal role in prenatal diagnosis, though more homogeneity in access criteria and a consensus on clinical management of controversial information management is envisageable to reach widespread use in the near future.

## 1. Introduction

**Figure 1:**
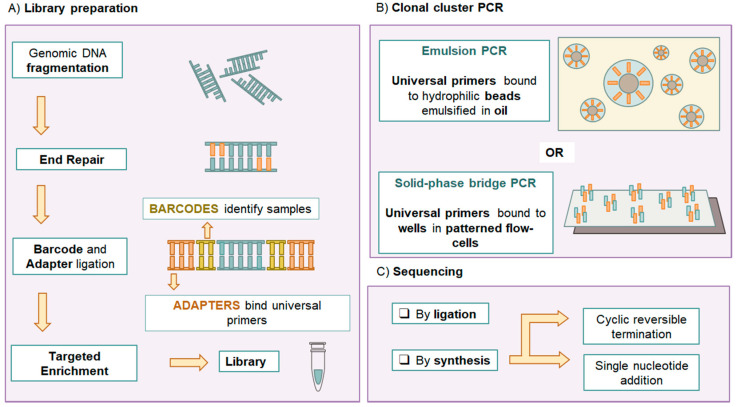
**Next Generation Sequencing (NGS) experiment workflow**. (**A**) Genomic DNA extracted from the samples is first processed in libraries. Purified genomic DNA is fragmented, then the fragments go through an end-repair phase and barcode and adapters ligation. Barcodes are unique sequences identifying specific samples within a run. Adapters are universal sequences used for PCR amplification and fragment capture. Some protocols require targeted enrichment (with PCR or biotin-streptavidin magnetic pulldown) to capture the desired fraction of the genome. (**B**) The libraries then undergo the crucial step of clonal cluster PCR amplification. This step leads to thousands of identical copies of the original DNA fragments in defined areas, allowing spatial resolution of localized, non-overlapping clonal clusters. This can be done with either emulsion-PCR, on hydrophilic beads emulsified in oil, or Solid-state bridge PCR, in the micro-wells of a patterned flow cell. (**C**) The sequencing reaction can be based on DNA ligase (sequencing by ligation) or DNA synthetase (sequencing by synthesis). The latter is the most common. Sequencing by synthesis can take place by Single Nucleotide Addition or by Cyclic Reversible Termination.

**Figure 2:**
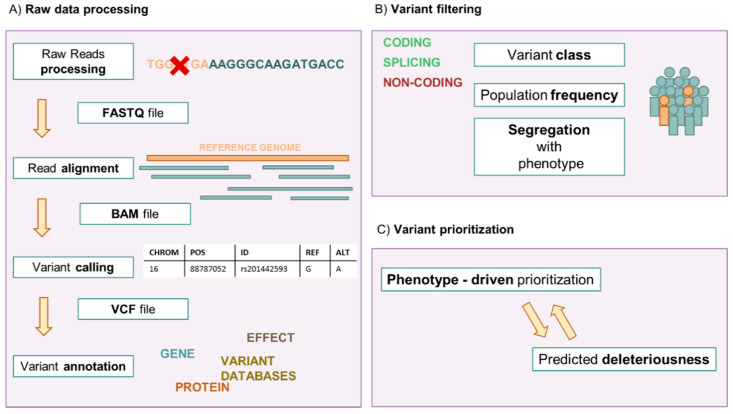
**NGS bioinformatic workflow.** (**A**) The sequencing reaction generates millions of short reads (40 to 400 nucleotides long). The reads are processed by marking duplicates and barcode and adapter sequences. Individual reads are retained in a FASTQ file. They are then aligned to the reference genome, generating a BAM file. Variants are identified (called) from nucleotide positions differing from the reference sequence, and gathered in a VCF file, consisting of a list of genomic coordinates with the reference sequences, the putative variants and quality scores. These variants are then annotated with information gathered from various databases, on variant frequency, gene(s) involved, gene products, predicted deleteriousness, reported pathogenicity or benignity. They are then manually analyzed by filtering (**B**) and prioritization (**C**). Variants with no impact on gene products, high frequency in the general population and not segregating with the phenotype are ruled out. The remaining variants are prioritized by various criteria, for example by potential relation to the phenotype or predicted deleteriousness.

Genetic testing in prenatal diagnosis is a precious tool providing valuable information in clinical management and parental decision making in a complex setting. Since the introduction of fetal karyotyping after amniocentesis, cytogenetic and molecular testing have evolved rapidly, and many genetic conditions can now be tested on fetal samples [1]. Next Generation Sequencing (NGS) has revolutionized clinical practice in Medical Genetics, becoming a staple of prenatal diagnosis [2]. The term NGS refers to a group of massive and parallel nucleic acid sequencing technologies. The techniques currently used in clinical practice are Second Generation Sequencing or Short Read Sequencing approaches [3]. The target region sequence is inferred by aligning multiple short individual partially overlapping reads to a reference sequence [3]. NGS approaches enable the sequencing of targeted gene panels or broader fractions of the genome with rapid turn-around time [4] (Figure 1 and Figure 2).

Exome Sequencing (ES) refers to the capture and sequencing of the coding fraction of the genome. When the test is designed to ideally capture the set of all coding exons, it can also be called Whole Exome Sequencing (WES) [3]. The rationale behind this is that 85% of known disease-causing mutations are exonic, but coding exons only account for about 1.5% of the genome [5]. Clinical Exome Sequencing (CES) or Targeted/Focused Exome Sequencing captures genes implied in Mendelian disorders [6]. This set of 5000–7000 genes, also called “Mendeliome,” is a dynamic entity, as research is still evolving [6]. Whole Genome Sequencing (WGS) refers to the unbiased sequencing of the genome, without targeted capture [3]. WGS yields the highest exon coverage, while also providing information on non-coding sequences and Copy Number Variation (CNV) imbalances [7]. However, we are far from understanding most of the non-coding variants and the bulk of sequencing data and bioinformatic analysis hinders a widespread WGS application. When performing WGS or WES/CES, the analysis can be initially limited to a given subset of genes, an in silico panel [8]. A key analytical parameter assessing the reliability of NGS data is “coverage”. “Depth” of coverage is the number of individual reads including a given nucleotide. “Average depth/coverage” is the average number of reads per position. “Breadth” of coverage is the targeted sequences percentage covered at a given depth [9].

Despite their undeniable strengths, NGS technologies do have challenges. Especially in capture-based methods, some regions may not be appropriately covered [7]. Unbalanced rearrangements are difficult to assess [10], single-exon deletions or duplications and CNV analysis, only performed on ES or WGS data, require dedicated algorithms and software, not all widely available [10].

A Variant of Uncertain Significance (VUS), incidental and secondary findings are also significant challenges for laboratories and clinicians [11,12]. Incidental findings are non-actionable findings independent from clinical indication. Secondary findings are variants from a specific American College of Medical Genetics and Genomics (ACMG) gene list, unrelated to the primary presentation and medically actionable. These difficulties can hamper genetic counseling, especially in prenatal diagnosis [11,12].

Currently, ES is extensively performed in post-natal practice, with a diagnostic yield of 25–45% [13,14]. Surprisingly, there are no worldwide guidelines for prenatal ES (pES) application, despite its increasing importance.

Diagnostic pES is usually performed as second- or third-line testing in fetuses with structural anomalies, non-diagnostic karyotype and chromosomal microarray (CMA), familial history and/or prenatal presentation suggesting monogenic disorders [15,16]. The role of NGS in non-invasive prenatal screening [17,18,19,20], or of other testing approaches, is outside the scope of this review.

Fetal structural anomalies occur in 2% of pregnancies [21]. Karyotype anomalies are found in 8%–10% of these cases, and CMA leads to a further 6–7% diagnostic yield [21,22]. Proper indications for pES are still debated, as well as the choice among WES, CES or other approaches. Given this variability, the diagnostic yield for pES still varies widely among studies [23,24].

The aim of this review is to evaluate pES diagnostic yield, inconclusive and negative-result rates, indications and challenges, while providing prompts for standardized practice and future directions.

## 2. Materials and Methods

We performed a Systematic Review of the literature on ES in prenatal diagnosis, according to the PRISMA guidelines [25]. We searched the PubMed database for ‘Exome’ AND ‘Prenatal’ (up to 12/30/2020). Titles and abstracts were examined. Papers not describing the application of pES were excluded. Then, individual articles were analyzed. Papers describing pES performed after a negative targeted panel were excluded. Case reports were excluded. Case-studies, reviews, seminars, descriptive studies, correspondences, editorials, commentaries, position statements and committee opinions were deemed eligible.

To investigate the diagnostic yield, we analyzed papers reporting multiple cases of diagnostic pES application during pregnancy or after pregnancy interruption. Papers describing access criteria for ES were retained, when clinical indication was primarily based on Ultrasound (US) anomalies. Papers providing recruiting criteria based on familial anamnesis were excluded, due to the possible bias. In all cases, karyotype and CNV analysis were inconclusive. Diagnoses achieved exclusively via karyotyping or CMA and pediatric diagnoses were excluded. We divided the selected cases in two groups based on recruitment criterion: the first one includes fetuses presenting any structural anomaly, regardless the involved organ, the second one presenting specific class anomalies. Cases were classified as positive (pES yielded a pathogenic/likely pathogenic variant explaining the phenotype), inconclusive (a VUS or variants in novel candidate genes were detected), and negative (no variant relevant to the phenotype). We calculated the diagnostic yield for each selected paper as (number of cases with pathogenic/likely pathogenic variant identified via pES)/(eligible cases). We scored the weighted average to make yield rates comparable despite the different sample numbers included in each article. We scored the rate of inconclusive results as (number of cases with a VUS or alleged novel candidate genes identified via pES)/(eligible cases). When available, we gathered negative results reported by authors. If this information was not provided, we calculated negative cases (regardless of incidental/secondary findings) by subtracting both positive and inconclusive results from the number of selected cases. We collected the number of incidental/secondary findings, but we did not score a specific rate as the number of consents gathered for these findings was seldom provided.

## 3. Results

**Figure 3:**
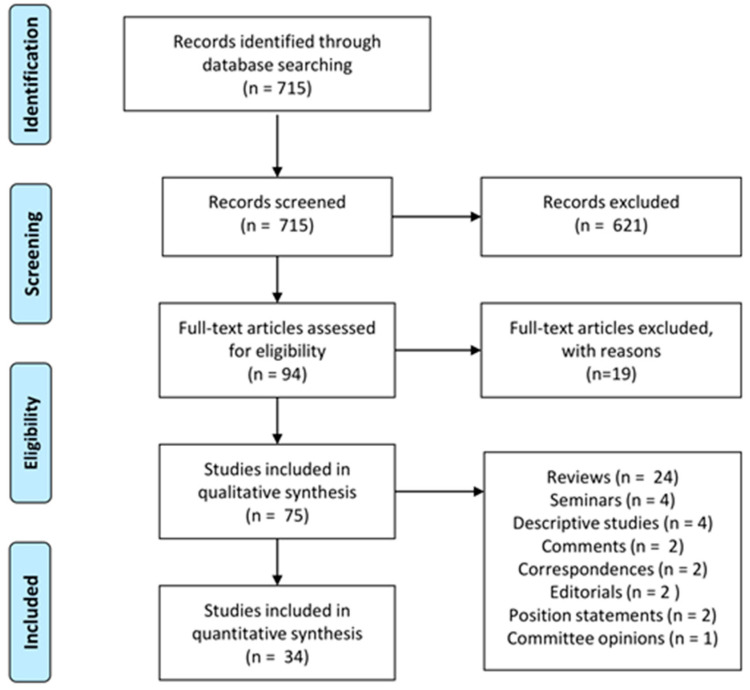
**PRISMA systematic review flow chart.** The chart illustrates the review process performed highlighting the number of papers identified, screened, elected and included in qualitative and quantitative synthesis.

The review process is summarized as a PRISMA flow chart in Figure 3. The initial search yielded 715 results. After the application of abstract examination criteria 94 papers were retained, resulting in: n. 53 case studies, n. 24 reviews, n. 4 seminars, n. 4 descriptive papers, n. 2 editorials, n. 2 comments, n. 2 correspondences, n. 2 position statements, n. 1 committee opinion.

Among papers reporting cases, n. 34 were deemed eligible for the analysis on pES diagnostic yield. n. 17 papers recruited fetuses reported as having any structural anomaly, either associated or isolated, with 1840 cases and 340 diagnoses.

n. 17 investigated specific single class anomalies, with 1421 cases and 217 diagnoses.

### 3.1. pES in Fetuses Selected for US Anomalies (Regardless of the Affected Organ)

We reviewed 17 (Table 1) articles scoring their diagnostic yield (range 8.5–47%; average 28%; weighted average 19%). Six papers reported secondary/incidental findings and 15 reported inconclusive results (average 16%; weighted average 12%). pES showed negative results for the test indication in an average of 56% of cases (weighted average 71%, calculated for the 15 articles reporting inconclusive findings), ranging from 26% to 88%. Five studies described less than 30 fetuses, while 12 papers involved more than 30 cases.

### 3.2. pES in Fetuses Selected for Specific Class Anomalies 

We selected 17 articles (Table 2). Seven papers described less than 30 samples, while 10 papers involved more than 30 cases. Five papers concerned congenital heart defects (CHD), 2 congenital anomalies of the kidney and urinary tract (CAKUT), 3 skeletal dysplasias, 3 central nervous system (CNS) malformations, 2 increased nuchal translucency (INT), 1 corpus callosum anomalies and 1 non-immune hydrops fetalis. The diagnostic yield ranged 6–92% (average: 32%; weighted average: 14%). Negative results ranged 8–95% (average: 57%; weighted average: 76%). Nine papers disclosed inconclusive results. Eleven papers reported incidental findings.

### 3.3. Other Studies

From the articles initially selected, some were subsequently excluded because they did not reflect our target, or due to the presence of bias that would make them not comparable to the yields of other papers.

We excluded:

- 30-case study, because they included both fetuses and neonates [60,61].
- 14-case study due to lack of inclusion criteria [62].
- 7-case study due to lack of clear eligibility criteria [63].
- 44-case study due to the higher a-priori risk for consanguinity and recurrence [64].
- 80-families study, in which they tested parents for recessive disorders [65].
- 73-samples study, because they did not present sufficient data to be compared to the other papers [66].
- 20-case study because it combined prenatal and postnatal phenotyping to interpret WES variants [67].
- 45-case study, Jewish descent, excluded due to the different inclusion criteria and the ethnicity at high risk for recessive disorders [68].
- 19-case study, for inhomogeneity in inclusion criteria and chromosomal anomalies/CNV assessment [69].
- 6-case study for inhomogeneity in inclusion criteria and chromosomal anomalies/CNV assessment [70].
- 102-case study, because 15 fetuses were elected for multiple anomalies highly suggestive of a genetic disorder, while further enrollment was extended to each pregnancy with fetal anomaly [71].
- 183-case study because it was designed to identify novel genes causing CAKUT [72].
- 30-case study, as the same cases were also included in a subsequent study [73].
- 56-case study, because pES was performed after a negative gene panel [74].
- 9-case study due to the unsystematic pES accession [75].
- 68-case study because they proposed a diagnostic algorithm for the Bardet-Biedl syndrome diagnosis, without presenting cases [76].
- 16-case study because they performed panel genes [77].
- 6-case study because it investigated a very specific phenotype after negative panel [78].
- 708-case study due to the postnatal diagnosis [79].

## 4. Discussion

### 4.1. pES Cohorts and Series Analysis

Despite a conspicuous literature on pES in single case reports, there are relatively few prenatal studies in which diagnostic yield, secondary/incidental and inconclusive results are explicit. Case reports reporting only successful tests are misleading, and impact on the perception of the pES efficiency.

The deceased fetuses described in some articles might introduce another bias due to the phenotypes severity, and their higher likelihood of genetic conditions. Comparing diagnostic yield in ongoing pregnancies and fetal demises would be useful, but this information is often impossible to infer.

A major challenge in prenatal diagnosis is represented by the evolving and incomplete development that limits defining phenotypes. Other limits might be the US-operator experience and undetectable features (e.g., developmental delay). Diagnostic strategies change over time further increasing heterogeneity if the observation period is fairly long.

The reasonably reassuring role of negative results has not been hinted at by some authors, but never scientifically ascertained [26].

Different centers, by serving different populations, might introduce significant selection or ascertainment biases in their works. A higher molecular diagnostic rate is expected in specialized institutions or inbred populations. For these reasons, we excluded the previously reported 19 studies from the review. Even papers deemed suitable for the analysis might present, to a lesser degree, the above-mentioned biases.

#### 4.1.1. Exome Sequencing in Fetuses Enrolled by US Anomalies (Regardless of the Affected Organ)

Diagnostic yield of these papers ranges from 9% to 47%, with an average of 28%, and a higher rate for fetuses showing multiple malformations.

The relatively high yield can be influenced by familial cases and consanguinity [33,35,36]. The diagnostic yield appears to be noticeably lower in large cohorts, and higher in small series with tight inclusion criteria. We scored a diagnostic rate weighted average of 19%. 

Several studies show a low pES yield for INT, but persisting INT or further anomalies might direct towards a molecular diagnosis.

Inconclusive findings are noted in 15 series (weighted average: 12%). Some authors decided to occasionally report inconclusive findings after collegial decisions. Others overcame the problem reporting only pathogenic/likely pathogenic variants in genes related to pES indications or known to cause significant disorders during childhood [32]. Normand et al. highlight that couples with a fetus diagnosed with US anomalies in which a VUS is detected require more support than families that receive a definitive diagnosis throughout pES [31]. 

Secondary/incidental findings were noted in six articles, a too low number to draw conclusions. However, this infrequent and inhomogeneous trend is suggestive.

#### 4.1.2. Exome Sequencing in Fetuses Selected for Specific Class Anomaly

Diagnostic yield ranged from 6% to 92%, with an average of 32%. The differences in investigated anomalies and sample numbers might account for the discrepancies. The diagnostic yield weighted average was 86% in Skeletal dysplasias, 44% in CNS anomalies, 11% in CHD, and 8% in INT cases. Skeletal dysplasias’ diagnostic yield was higher if compared to the rate of larger studies for the same anomalies [37,38]. This can be accounted for by tighter selection criteria. Sample numbers for CNS anomalies were too poor to provide assumptions.

The most solid predictions can be made for CHD, with an 8%-13% diagnostic yield consistent among vast cohorts ranging from 197 to 610 samples [37,38,49,50,51].

Inconclusive, secondary/incidental findings and negative results do not allow proper comments and comparisons due to marked inhomogeneity.

### 4.2. Recent Past and State of the Art

NGS has revolutionized clinical practice in medical genetics, with the unprecedented ability to rapidly analyze large sets of genes. Therefore it is having a significant impact on both genetic research and clinical diagnostics. The first prenatal NGS-diagnosis was performed via WGS on a fetus with multiple anomalies and a de novo translocation, disrupting *CHD7*, resulting in CHARGE syndrome [80]. pES was initially limited to the research field [81,82], and regarded as a tool for the discovery of novel disease-causing genes in familial cases with lethal phenotypes [81]. Its world-wide application in selected cases led to many discoveries and reports, expanding the prenatal presentation and phenotypic spectrum of many genetic conditions. No assumption about a putative widespread clinical application of pES could be made, as candidate cases were selected for high probability of monogenic disorder, and there was a possible positive-result publication bias. The transition to the diagnostic setting began swiftly, encouraged by the very high yield (25%) inferred from postnatal cases [13]. Patients eligible for postnatal ES usually display a more overt phenotype, comparably easier to dissect and investigate. The deeper phenotyping available in the post-natal setting confers stronger clinical suspicion and a solid base for molecular analysis, and can grant a higher diagnostic yield.

The enthusiasm raised by the initial reports and cohorts collided with the critical issues, typical of prenatal diagnosis. pES application in fetuses with structural anomalies but no family history suggestive for monogenic disorders leads to a considerably smaller amount of positive results [67], many interpretative challenges, and difficulties in counseling and decision-making. The two largest pES studies reported an incremental diagnostic yield of 8.5% [37] and 10% [38], and an impressive ambiguous result rate up to 20%. Despite being below expectations, the incremental yield of pES is at least comparable to that of karyotype as a first-tier test or CMA as a second-tier test.

Current practice is still evolving and guidelines are not well established. The first inter-society position statement providing a framework for pES [83], discouraged routine use of pES, and suggested its use mainly in research, with prompts for single-case evaluation. More recently, the ACMG published a statement on pES [84]. This document covers a wide array of topics, from pre-test counseling to reanalysis after negative results, providing much needed prompts on debated questions like reporting a VUS, incidental and secondary findings. However, it has not to be considered a binding guideline, being interpretable variously in each case.

At present, we have solid indications for pES based on familial anamnesis, with great results in research and clinical settings alike, offering pES as a first-tier routine test is strongly discouraged, and there is a vast grey zone encompassing all fetuses with structural anomalies but non-conclusive karyotype and CMA.

### 4.3. Present Challenges

Defining homogeneous criteria for reporting variants prenatally is a significant challenge. In most countries, prenatal molecular diagnosis of a known mutation is acceptable only for variants classified as pathogenic/likely pathogenic according to ACMG guidelines [85].

In pES, the likelihood of detecting a VUS increases and the question of whether a VUS should be reported or not has not been resolved. Standardized guidelines are not available, resulting in inhomogeneous conducts, even within the same country. In the last “Points to consider document” [84], ACMG highlights how laboratories currently have different approaches to report a VUS prenatally, rather recommending to define clear policies.

The first point to clarify concerns a VUS in genes related to the fetal phenotype. The benefit/risk ratio should be carefully assessed, since a VUS identified in an indication-relevant gene might impact current and/or future pregnancies [86], but uncertainties may cause parental anxiety without immediate benefit for decision-making [87]. ACMG suggests reporting a VUS in phenotype-fitting genes, especially for autosomal recessive conditions if a VUS is found in “trans” with a pathogenic/likely pathogenic variant [84]. Inconclusive results could be also linked to a variant in a candidate gene (gene with limited evidence of disease association). The choice of reporting this kind of variant is currently ascribed to the single laboratory policy. ACMG statement suggests the classification of a variant in a candidate gene no higher than a VUS and recommends to clearly specify the policy during the consent process [84]. 

In our review, inconclusive results were 12% in the first group, a relevant percentage in comparison to the total diagnostic yield. It should be considered that variant classification may evolve over time as new evidence emerges [87]. In this perspective, postnatal sequencing data access is appealing and underestimated. Fetal results are normally included in the mother’s records, making data-retrieving difficult for child follow-up [88]. It has been suggested that pES data reanalysis should be considered if a new phenotype emerges after birth or a future pregnancy is planned, but the exact time has not been clarified yet [86]. In pediatric populations, ES data re-analysis has been observed to increase the diagnostic yield after 12 months [89]. It is also debated if clinicians or laboratories should be primarily responsible for such re-analysis [15,86].

More complex problems concern variants unrelated to the fetal indication, such as secondary/incidental findings. For postnatal ES secondary findings, the ACMG recommends to report pathogenic/expected pathogenic variants in 59 actionable genes, causing highly penetrant disorders for which interventions preventing/reducing morbidity and mortality are available [90]. The previous ACMG statement (56 genes) recommended to include children, explicitly excluding fetuses [91]. A subsequent revised document suggested to offer an opt-out option after pre-test counseling [92]. The last ACMG statement confirms the opt-out option, recommending to report secondary findings if parents consented [84]. However, it should be noticed that the expression of consent by parents might prevent the future individual possibility from declining to receive such results [87]. In addition, a survey on genetics professionals’ attitudes towards pES, highlighted that for most participants the definition of “actionable results” is different in the prenatal setting due to the option for termination [93].

Fetal incidental findings actually include variants unrelated to primary test indication that are not included on the ACMG secondary findings list, such as genes causing neurodevelopmental phenotypes not detectable by fetal US [84]. Currently, ACMG recommends to report only high-penetrant pathogenic variants known to cause moderate/severe childhood onset disorders [84].

Additional findings such as non-paternity or consanguinity are also detectable in pES, and parents should be aware before testing is undertaken that these may be identified [87].

In trio analysis, secondary/incidental findings may be searched for and detected in the parental as well as in the fetal genotypes. These results could provide couples and families with unwanted information [82]. ACMG statement [84] recommends laboratories to establish clear policies regarding whether incidental findings report is limited to fetal variants or parents are also included (irrespective of fetal inheritance). Of note, the laboratory’s analysis process could be set to limit the assessment of parental variants to only those present in the fetus.

In our literature analysis, secondary/incidental findings were reported to families in a minority of cases only, probably reflecting the difficulties to manage such results [94]. In addition, it should be borne in mind that pES is usually proposed for US single or multiple anomalies, couples already presenting apprehension for the possible conditions underlying these anomalies [94]. They often lack sufficient time to reflect about the potential amount of information available after pES and to elect how much they want to know. Reporting inconclusive or incidental findings in this context is extremely delicate, and offering psychological support should be considered [95].

The importance of an adequate consent administration is a long-present concern in prenatal diagnosis and it represents a crucial point in the pre-test counseling [96]. In the era of prenatal genomics, additional issues emerge, due to the unprecedented quantity of detailed genetic data to be reported or retained, as discussed above.

The consent process requires to inform patients and to reach a good understanding of advantages and disadvantages before decision [12]. Professionals should provide available and manageable information [11,97] to consultants from all educational and cultural backgrounds [98]. This is not always feasible, as for couples with low school-education, for whom also the very concept of DNA could be difficult to manage. Returning much information after pES could influence decision-making about pregnancy termination [98] or the parents’ attitude towards their child [12], being informed about the risk of developing signs or symptoms of a genetic condition.

Incertitude about disease severity, the probability of presenting some symptoms or not and the emotional tool of a genetic condition are always present [99]. This incertitude may be increased in prenatal diagnosis and should be clearly presented to parents [95,99].

The significance of a negative result should be better investigated. It could be reassuring during pregnancy, but the “temporality” should be clarified beforehand, due to the evolving fetal phenotype and possible postnatal appearance of further anomalies.

### 4.4. Future Perspectives

This systematic review highlighted current evidence on advantages and disadvantages of pES. Major challenges emerged with an urgent need for specific guidelines.

A possible approach to reduce interpretative and ethical issues is to report only variants related to the specific indication or pregnancy-/delivery-relevant conditions or suggesting fetal therapies [100]. In this perspective, parents could opt for a secondary report to disclose findings relevant to postnatal care [88].

In the absence of homogeneous directions, we consider a deep discussion between clinicians and laboratory professionals essential, possibly in a collegial discussion, before reporting inconclusive findings. In our experience, the discussion on a VUS potentially phenotype-relevant is crucial, eventually reporting it with accurate explanation. Still, we deem reporting a VUS without such evidence self-defeating and really stressful for couples. The possibility of reassessing the pathogenicity of a variant with further imaging diagnostics or biochemical analysis performed on fetal or maternal samples (“genotype-first” approach) is intriguing but has not been sufficiently investigated.

The possibility of exome data re-analysis is stimulating, along with newborn follow-up. This approach requires NGS data accessibility after birth, and coordination between professionals to avoid repeating genetic tests. Different approaches have been proposed even by ACMG, such as periodical reanalysis of negative results, or targeted re-analysis for cases with evolving phenotype [84]. Periodical re-analysis requires significant organization efforts and world-wide guidelines to produce homogeneous data. Thus, we cautiously suggest performing pES re-analysis primarily based on phenotypic evolution.

To optimize pES reliability, reducing interpretative issues, we endorse the future delineation of a “Prenatalome,” a consensus set encompassing genes confidently linked to fetal phenotypes to be exclusively analyzed, possibly as an in silico panel. Gene panels specifically designed for prenatal diagnosis have already been proposed and widely applied [36,37] and also available in open-source platforms, such as PanelApp [101].These panels are, however, not uniformly used in prenatal diagnosis, and their application furtherly distances the advisable homogeneity required in such a complex setting. Ideally, the definition of a set of genes for prenatal analysis and a sub-set for secondary findings could lead to reliable, homogeneous data for clinical diagnoses and scientific investigations.

To reach this level of phenotype-genotype correlation a deep fetal phenotyping [102] would be advisable, standardized information about prenatal phenotypes is still scarce, and pES will inevitably expand prenatal phenotypes not previously described [103].

Human Phenotype Ontology (HPO) descriptors are widely used to standardize clinical information for molecular analysis in postnatal diagnosis [104]. To date, around 150 HPO terms describe prenatal development abnormalities, but specific issues should be taken into consideration. For example, fetal presentations may differ from postnatal signs classically associated with the same genetic condition. These signs could also evolve or resolve during pregnancy, and terms to describe the “worsening” or “offset” of phenotypic features are also needed. A further point is that prenatal information comes from different imaging methods, such as US or Fetal MRI [104]. These imaging methods offer indirect evaluation for anatomy and function, and HPO terms have to be extended to this kind of modalities. A specific prenatal phenotype HPO project has been recently announced [105]. In addition, algorithms, software and bioinformatic tools should be specifically recalibrated for fetal signs, and fetal variant databases, equivalent to postnatal databases like ClinVar or the Human Genetics Mutation Database (HGMD), created [98]. 

## 5. Conclusions

pES represents an extraordinary tool to better understand the genetic causes of fetal anomalies. We showed that diagnostic yield largely varies between different cohorts, due to differences in selection criteria, methods of analysis, specific anomaly/ies investigated and sample numbers. We noticed a great inhomogeneity in pES application, with many uncertainties and a lack of univocal position statements. An international effort is needed to define more detailed criteria for pES application and analysis, variants interpretation and report.

## Figures and Tables

**Table 1:** pES in fetuses selected for US anomalies (regardless of the affected organ).

Article	Test	Geographic Area	Diagnostic Yield (%)	Inconclusive Findings (%)	VUS (%)	Novel Candidate Genes (%)	Negative (%)	Secondary/ Incidental Findings
Pangalos, 2016 [26]	in silico panel from WES data	Greece	6/14 (43%)	1/14 (7%)	not provided	not provided	7/14 (50%)	not provided
Vora, 2017 [27]	in silico panel from WES data	USA	7/15 (47%)	3/15 (20%)	2/15 (13%)	1/15 (7%)	5/15 (33%)	not provided
Yates, 2017 [28]	WES	USA	17/84 (20%)	45/84 (54%)	38/84 (45%)	7/84 (9%)	22/84 (26%)	2
Boissel, 2018 [29]	WES	Canada	19/101 (19%)	5/101 (5%)	1/101 (1%)	4/101 (4%)	77/101 (76%)	not provided
Fu, 2018 [30]	WES	China	47/196 (24%)	25/196 (13%)	25/196 (13%)	0	124/196 (63%)	12
Leung, 2018 [31]	WES	China	3/33 (9%)	6/33 (18%)	6/33 (18%)	0	24/33 (73%)	not provided
Normand, 2018 [32]	WES	USA	46/133 (35%)	not provided	not provided	not provided	---	not provided
Meier, 2019 [33]	WES	Switzerland	11/26 (42%)	3/26 (12%)	0	3/26 (12%)	12/26 (46%)	3
Daum, 2019 [34]	WES	Israel	16/77 (21%)	not provided	not provided	not provided	---	not provided
Quinlan-Jones, 2019 [35]	in silico panel from WES data	UK	10/25 (40%)	6/25 (24%)	6/25 (24%)	0	9/25 (36%)	not provided
De Koning, 2019 [36]	WES	Netherlands	8/20 (40%)	0/20 (0%)	0	0	12/20 (60%)	3
Lord, 2019 [37]	WES	UK	52/610 (9%)	24/610 (4%)	24/610 (4%)	0	534/610 (88%)	not provided
Petrovski, 2019 [38]	WES	USA	24/234 (10%)	46/234 (20%)	not provided	not provided	164/234 (70%)	4
Becher, 2020 [39]	WES	Denmark	9/35 (26%)	7/35 (20%)	7/35 (20%)	0	19/35 (54%)	1
Chen, 2020 [40]	CES	China	20/105 (19%)	12/105 (11%)	12/105 (11%)	0	73/105 (70%)	not provided
Dempsey, 2020 [41]	CES	UK	18/52 (35%)	13/52 (25%)	13/52 (25%)	0	21/52 (40%)	not provided
Qi, 2020 [42]	CES	China	27/80 (34%)	5/80 (6%)	5/80 (6%)	0	48/80 (60%)	not provided

**Table 2:** pES in fetuses selected for specific class anomalies.

Article	Anomaly	Test	Geographic Area	Diagnostic Yield (%)	VUS (%)	Novel Candidate Genes (%)	Negative (%)	Secondary/ Incidental Findings
Weitensteiner, 2018 [43]	Brain malformations	WES	Germany	3/6 (50%)	0	0	3/6 (50%)	1
Westphal, 2019 [44]	Congenital heart diseases	CES	Germany	6/30 (20%)	2/30 (7%)	2/30 (7%)	20/30 (67%)	3
Yang, 2019 [45]	Skeletal dysplasias	CES	China	6/8 (75%)	0	0	2/8 (25%)	not provided
Sun, 2020 [46]	Congenital cardiac left-sided lesions	WES	China	13/66 (20%)	5/66 (8%)	6/66 (9%)	42/66 (64%)	1
Heide, 2020 [47]	Corpus callosum abnormalities	WES	France	12/62 (19%)	6/62 (10%)	0	44/62 (71%)	not provided
Lei, 2020 [48]	Congenital anomalies of the kidney and urinary tract	WES	China	12/163 (12%)	2/163 (1%)	0	149/163 (91%)	9
Li R., 2020 [49]	Congenital heart disease	WES	China	26/260 (10%)	16/260 (6%)	16/260 (6%)	202/260 (78%)	7
Mone, 2020 [50]	Congenital heart disease	in silico panel from WES data	UK	25/197 (13%)	10/197 (5%)	0	162/197 (82%)	not provided
Qiao, 2020 [51]	Congenital heart disease	WES	China	24/300 (8%)	32/300 (11%)	0	244/300 (81%)	48
Sparks, 2020 [52]	Non-immune hydrops fetalis	WES	USA	37/127 (29%)	12/127 (9%)	0	78/127 (61.41%)	4
Tan, 2020 [53]	Brain anomalies	CES	China	5/11 (45%)	0	0	6/11 (55%)	not provided
Tang, 2020 [54]	Skeletal dysplasias	WES	China	6/8 (75%)	0	0	2/8 (25%)	not provided
Xue, 2020 [55]	Increased nuchal translucency	WES	China	3/24 (13%)	0	0	21/24 (88%)	2
Yang, 2020 [56]	Increased nuchal translucency	CES	China	4/73 (6%)	0	0	69/73 (95%)	7
Zhou, 2020 [57]	Congenital anomalies of the kidney and urinary tract	WES	China	3/41 (7%)	0	0		1
Han, 2020 [58]	Skeletal dysplasias	CES	China	24/26 (92%)	0	0	2/26 (8%)	not provided
Li L., 2020 [59]	Cerebellar vermis defects	WES	China	8/19 (42%)	2/19 (11%)	0	9/19 (47%)	1

## Data Availability

The data presented in this study are available in the articles cited in the Reference Section.
